# Stimulation of the Salicylic Acid Pathway Aboveground Recruits Entomopathogenic Nematodes Belowground

**DOI:** 10.1371/journal.pone.0154712

**Published:** 2016-05-03

**Authors:** Camila Cramer Filgueiras, Denis S. Willett, Alcides Moino Junior, Martin Pareja, Fahiem El Borai, Donald W. Dickson, Lukasz L. Stelinski, Larry W. Duncan

**Affiliations:** 1 Department of Entomology, Universidade Federal de Lavras, Lavras, MG, Brazil; 2 Entomology and Nematology Department, Citrus Research and Education Center, University of Florida, Lake Alfred, Florida, United States of America; 3 Plant Protection Department, Faculty of Agriculture, Zagazig University, Sharkia, Egypt; 4 Departamento de Biologia Animal, Instituto de Biologia, Universidade Estadual de Campinas - UNICAMP, Campinas, SP, Brazil; 5 Entomology and Nematology Department, University of Florida, Gainesville, Florida, United States of America; United States Department of Agriculture, Beltsville Agricultural Research Center, UNITED STATES

## Abstract

Plant defense pathways play a critical role in mediating tritrophic interactions between plants, herbivores, and natural enemies. While the impact of plant defense pathway stimulation on natural enemies has been extensively explored aboveground, belowground ramifications of plant defense pathway stimulation are equally important in regulating subterranean pests and still require more attention. Here we investigate the effect of aboveground stimulation of the salicylic acid pathway through foliar application of the elicitor methyl salicylate on belowground recruitment of the entomopathogenic nematode, *Steinernema diaprepesi*. Also, we implicate a specific root-derived volatile that attracts *S. diaprepesi* belowground following aboveground plant stimulation by an elicitor. In four-choice olfactometer assays, citrus plants treated with foliar applications of methyl salicylate recruited *S. diaprepesi* in the absence of weevil feeding as compared with negative controls. Additionally, analysis of root volatile profiles of citrus plants receiving foliar application of methyl salicylate revealed production of d-limonene, which was absent in negative controls. The entomopathogenic nematode *S. diaprepesi* was recruited to d-limonene in two-choice olfactometer trials. These results reinforce the critical role of plant defense pathways in mediating tritrophic interactions, suggest a broad role for plant defense pathway signaling belowground, and hint at sophisticated plant responses to pest complexes.

## Introduction

Plants adopt constitutive and induced strategies to defend against herbivores and pathogens both aboveground and belowground [1, 2]. These defenses can act directly against the offending herbivore, producing or releasing toxins that deter feeding behavior [3]. Indirectly, these defenses can result in the release of herbivore induced plant volatiles that recruit natural enemies [3]. These tritrophic interactions involving recruitment of natural enemies have been observed aboveground [4, 5] and belowground where feeding by larvae of *Diabrotica virgifera virgifera* results in release of *E*-*β* caryophyllene and recruits the entomopathogenic nematode *Heterhorabditis megidis* [6]. Similarly, in citrus, feeding belowground by larvae of the weevil *Diaprepes abbreviatus* results in release of pregeijerene which recruits a wide variety of nematodes, including entomopathogenic nematodes that are natural enemies of *D. abbreviatus* [7–9].

These tritrophic interactions between plants, herbivores, and their natural enemies above and belowground are mediated by stimulation of defense pathways within plants [3]. Stimulation of these plant defense pathways can occur through herbivory [10], plant-to-plant communication [11], or application of chemicals that elicit plant defense responses [12]. Among a myriad of plant defense pathways, a prominent pathway that has important roles in plant defense against both pathogens and herbivores is the salicylic acid pathway [13, 14]. It is so called because of the prominent role salicylic acid plays in stimulating plant defense and its known role in recruiting natural enemies aboveground [15].

In addition to its role in recruiting natural enemies aboveground, the salicylic acid pathway also mediates interactions between herbivores and pathogens. Stimulation of the salicylic acid pathway through synthetic elicitors can reduce bacterial lesion development [16] and can affect plant resistance to herbivores [17]. In addition, the sequence of induction can have ramifications for plant defense pathway stimulation and herbivore-pathogen resistance [16, 18]. Multiple stimulation of plant defense pathways also has tritrophic effects on natural enemies aboveground [19].

Less is known regarding the role the salicylic acid plant defense pathways play in mediating plant responses belowground. While stimulation of plant defenses aboveground has effects belowground, and vice versa, the dynamic nature of plant defense pathways in mediating this communication between the terrestrial and subterranean environments is less well understood [20–22]. Effects of plant defense stimulation aboveground on interactions belowground are varied and occasionally nonexistent [1, 22, 23]. Similarly, the role of plant defense pathways in stimulating production of herbivore induced plant volatiles for the recruitment of natural enemies belowground is not well understood.

Here, we explore the effect of stimulating the salicylic acid pathway aboveground on recruitment of natural enemies belowground. To do so, we applied an elicitor, methyl salicylate, to the leaves of citrus seedlings while monitoring the response of the entomopathogenic nematode *Steinernema diaprepesi* belowground both in the presence and absence of the larval weevil herbivore *D. abbreviatus*, a prominent polyphagous root pest of citrus and many other crops. The entomopathogenic nematode, *S. diaprepesi*, may be the most effective natural enemy of this polyphagous root herbivore and therefore we focused on this particular nematode as part of our multi-trophic investigation [24, 25].

## Materials and Methods

To evaluate the effect of plant defense pathway stimulation on recruitment of natural enemies belowground, particularly in the case of the salicylic acid pathway, 30*mL* of 130*μl*/*L* methyl salicylate was applied to the aboveground portion of citrus seedlings while nematode response was monitored in olfactometer bioassays belowground. Based on the nematode response, volatiles were collected from the roots of treated and control plants. Volatiles unique to treated plants were then evaluated for activity in two-choice bioassays.

### Organisms

Response of the infective juvenile stage of the entomopathogenic nematode *Steinernema diaprepesi* to 20 cm citrus Swingle Citrumelo (*Citrus paradisi* Macf. × *Poncirus trifoliata* L. Raf.) seedlings was evaluated in four-choice olfactometers. *S. diaprepesi* infective juveniles were originally collected from sentinel *D. abbreviatus* larvae in Florida citrus groves and then reared on *Galleria mellonela* larvae and collected on White traps [26, 27]. *S. diaprepesi* infective juveniles were maintained in shallow tissue culture flasks at 14°C and were used within two weeks after emergence. Fifth instar *D. abbreviatus* larvae used in methyl salicylate bioassay trials were reared on artificial diet from eggs laid by adults collected from Florida citrus groves [28, 29].

### Methyl Salicylate Bioassays

The attraction of the entomopathogenic nematode *S. diaprepesi* to citrus seedlings treated with foliar applications of elicitors in the presence and absence of belowground herbivory by *D. abbreviatus* larvae was evaluated in four-choice olfactometers (similar to six-choice olfactometers used for evaluating nematode behavior [6]) filled with clean autoclaved sand adjusted to 12% moisture by volume. Four-choice olfactometers were constructed from 4×4×4 inch (10.16 × 10.16 × 10.16cm) containers (Tupperware Corporation, Orlando, FL) perforated on each of the four sides to accomodate 2 inch (5.08cm) PVC pipe elbows. Connections were sealed with insulation and one citrus seedling was placed in each of the elbows. After allowing 48 hours for acclimatization, plants were treated with elicitor sprays. In each four-choice olfactometer, two opposing seedlings received treatment with methyl salicylate (MeSA) and two opposing seedlings were left as untreated, negative controls. Methyl salicylate treated seedlings each received 30*mL* of 130*μl*/*L* methyl salicylate (Sigma; CAS:119-36-8) by foliar spray in a Tween 20 and ethanol solution at 0.1 and 2.5*mL*/*L* respectively. Control seedlings did not receive the elicitor, only the Tween 20 and ethanol solution. For experiments involving *D. abbreviatus* herbivory, five approximately five week old *D. abbreviatus* larvae were placed directly on the roots of methyl salicylate treated and control seedlings. Forty-eight hours after application of the elicitors, approximately 2500 *S. diaprepesi* infective juveniles were released into the center of the olfactometer. After an additional 24 hours, nematodes were extracted from the responding arms using sugar centrifugation, then counted [30].

### Volatile Collection and Analysis

To investigate the potential role of volatile-mediated nematode attraction in the four arm olfactometers, volatiles were collected from the root systems of untreated citrus seedlings and seedlings treated with methyl salicylate. Volatiles were collected 48 hours after application of elicitors for one hour onto 30*mg* HayesepQ adsorbent filters (Volatile Assay Systems; VAS) at a flow rate of 160*ml*/*min*. Extracted volatiles were eluted off of the collection filters with two aliquots of 75*μl* methylene chloride. Five microliters of 1.5*μg*/*μl* nonyl acetate was added as an internal standard. A one microliter aliquot of each sample was then injected onto a Clarus 500 gas chromatograph—mass spectrometer (PerkinElmer, Waltham, MA) containing a 30*m* × 0.25*mm*−*ID* DB-5 capillary column. The column was held at 35°C for 3 minutes after injection and then increased 10°C per minute until reaching 260°C where it remained for an additional five minutes. Helium was used as a carrier gas at a flow rate of 2 ml per minute. Electron ionization spectra were compared with references found in the NIST Mass Spectral Library (2008) and then confirmed with available standards. Differences in volatile profiles between treated and control plants were examined and quantified by comparison to the nonyl-acetate internal standard.

### Volatile Bioassays

To investigate whether d-limonene, primarily responsible for the differences between volatile profiles of methyl salicylate treated and untreated control plants (see Results), may attract *S. diaprepesi*, two-choice sand-filled assays consisting of inverted 1.5 inch (3.81 cm) diameter PVC T-Tubes, capped on each end, were used. Individual assay tubes were filled with clean autoclaved sand adjusted to 12% moisture by volume after placing filter paper treated with either a blank control, 10*μl* of water, or 10*μl* aliquots of doses of d-limonene in water for a total of 17*ng*, 170*ng*, 1.7*μg*, *or*17*μg* at opposing ends of the olfactometer. Approximately 2000 *S. diaprepesi* infective juveniles were applied to the central orifice of each olfactometer. After 24 hours, responding nematodes were extracted from the sand in each PVC cap using Baermann funnels and counted [31].

### Statistical Analysis

*S. diaprepesi* infective juvenile response to salicylate-treated citrus plants in four-choice olfactometers was summed within each replicate for each treatment to avoid aggregation effects then examined for normality by visual inspection with quantile-quantile plots and Shapiro-Wilk’s test. Wilcoxon signed rank tests were then used to evaluate preference. Differences in volatile profiles between treated and control plants were quantified through comparison to internal standards. Mean quantities of collected volatiles were calculated and bootstrapped to determine 95 percent confidence intervals. *S. diaprepesi* infective juvenile preference for doses of d-limonene in two-choice olfactometers was evaluated by determining the percentage of infective juveniles responding to d-limonene in each replicate for each dose. Preference percentages were examined for normality through visual inspection with quantile-quantile plots and interrogation with Shapiro-Wilk’s test and subsequently evaluated for differences from a 50% response of no preference through one-sided t-tests with Bonferroni correction (reported as *p*_*adj*_). Data were collated in Microsoft Excel 2011 and analyzed using R version 3.2.2 [32] in the R Studio version 0.99.484 development environment [33]. Analysis was facilitated using the packages *xlsx* [34] for interface with Microsoft Excel, *tidyr* [35] and *dplyr* [36] for data arrangement and summary statistics, *ggplot*2 [37] for graphics capabilities, and *scales* for visual representation of scaling [38].

## Results

### Methyl Salicylate Bioassays

**Fig 1:**
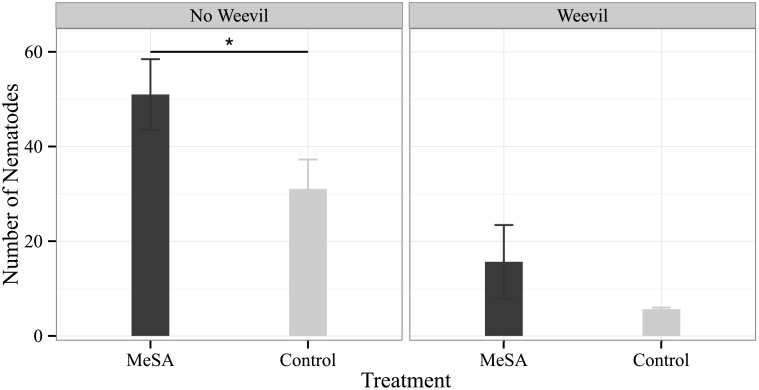
*S. diaprepesi* attraction to methyl salicylate (MeSA) treated citrus seedlings. Entomopathogenic nematode *S. diaprepesi* infective juvenile response to citrus seedlings treated aboveground with methyl salicylate in four-choice sand filled olfactometers both in the presence and absence of belowground herbivory by *D. abbreviatus* weevil larvae (n = 21). Bars and error bars denote mean number of respondents and standard error respectively. *S. diaprepesi* infective juveniles significantly preferred plants treated with methyl salicylate (MeSA) over control plants in the absence of weevil feeding damage.

The infective juveniles of the entomopathogenic nematode *S. diaprepesi* significantly (p = 0.01) preferred (27.7%; 95% Confidence Interval: 16.4%, 38.9% difference) plants treated with methyl salicylate (MeSA) over control plants in the absence of a weevil pest (Fig 1). Data were non-normal by visual inspection and interrogation with the Shapiro-Wilk normality test (W = 0.83, p = 0.004). In the presence of belowground feeding by the insect herbivore *D. abbreviatus* on both the control and treated plants, methyl salicylate treated plants were not significantly (p = 0.25) more attractive than controls (Fig 1).

### Volatile Collection and Analysis

**Fig 2:**
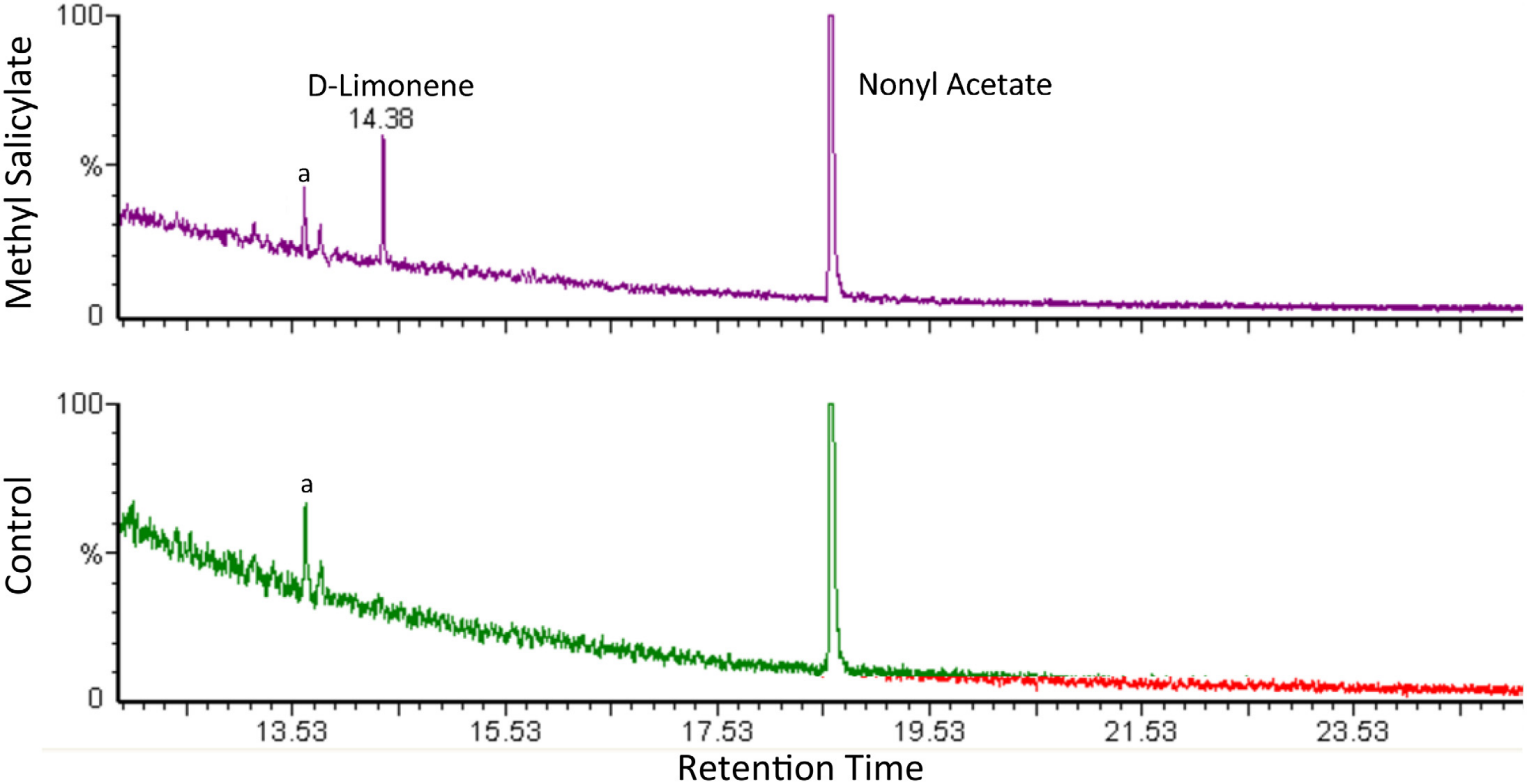
Volatile Profiles of Methyl Salicylate Treated and Control Plants. Sample chromatograms with volatile profiles of methyl salicylate treated (above) and control (below) plants. d-limonene (retention time 14.38; from 0.04 to 2.22ng) was present in treated plants, but not in controls (n = 10). Nonyl acetate was used as an internal standard. Decane (a) was also recovered in both standards and controls.

d-Limonene (retention time 14.38) was present in root volatile profiles of methyl salicylate treated plants but not detectable in the controls (Fig 2). An average of 0.61*ng*/*μl* (from 0.04 to 2.22*ng*/*μl*) d-limonene was detected in eluted samples from methyl salicylate treated plants; total amount of volatile d-limonene collected averaged 91.5*ng*.

### Volatile Bioassays

**Fig 3:**
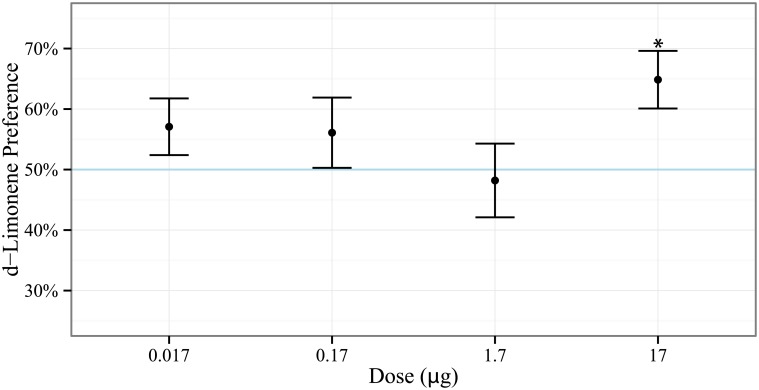
*S. diaprepesi* preference for d-limonene. Entomopathogenic nematode *S. diaprepesi* infective juvenile preference for doses of d-limonene as evaluated in two-choice sand filled olfactometers (n = 48). 50% response (horizontal blue line) indicates no preference. Points and error bars denote mean and standard error respectively. *S. diaprepesi* significantly preferred d-limonene at doses of 17*μg*.

Entomopathogenic nematode *S. diaprepesi* infective juveniles significantly (*p*_*adj*_ = 0.02) preferred d-limonene at doses of 17*μg* in two-choice olfactometer assays as compared with negative controls (Fig 3). Data were not significantly different from normal by visual inspection with quantile-quantile plots and interrogation with the Shapiro-Wilk test (p>0.28). Preferences for d-limonene at other doses were not significantly different from 50% (*p*_*adj*_ > 0.32).

## Discussion

Stimulation of the salicylic acid pathway through aboveground application of methyl salicylate resulted in recruitment of the entomopathogenic nematode *S. diaprepesi*. Herbivory by larvae of the weevil *D. abbreviatus* attenuates this response. Attraction in the absence of the weevil herbivore is likely mediated by belowground root release of the volatile d-limonene. This result suggests that insect larval feeding may induce a competitive plant defense response belowground.

These results highlight, for what we believe to be the first time, the direct role of the salicylic acid pathway in releasing induced plant volatiles for the recruitment of entomopathogenic nematode natural enemies belowground. While previous work has shown that herbivory belowground by the weevil *D. abbreviatus* can induce production of pregeijerene and attract entomopathogenic nematodes [8], the effects of stimulating the salicylic acid pathway on recruitment of subterranean natural enemies suggests a broader role for plant defense signaling for belowground natural enemies of herbivores.

This signaling serves little purpose if no receiver perceives the stimulus. The response of entomopathogenic nematodes to the d-limonene cue suggests that the entomopathogenic nematodes in this system are highly attuned to the volatiles in their environment. Entomopathogenic nematodes have been shown to respond to herbivory in connection to a variety of plant and herbivore species and to a variety of induced host plant volatiles belowground (e.g., *E*-*β* caryophyllene and pregeijerene) [6, 8, 39]. In previous work, however, such induced host plant volatiles were produced through herbivory or mechanical damage of a potential host. In our case, the d-limonene cue was released after stimulation of the salicylic acid pathway aboveground and in the absence of weevil herbivory. Interestingly, d-limonene is a terpene related to belowground signals indentified in earlier work [6, 8]. This may provide a different and complementary information pathway for plant defense belowground and does not simply signal presence of a host herbivore feeding on the roots.

Indeed, feeding by the weevil herbivore seemed to attenuate the response of belowground entomopathogenic nematodes. In the absence of salicylic acid pathway stimulation, herbivory by *D. abbreviatus* on Swingle Citrumelo citrus seedlings recruits entomopathogenic nematodes through release of the herbivore-induced volatile pregeijerene within twenty-four hours [8]. In the absence of herbivory, salicylic acid pathway stimulation recruited entomopathogenic nematodes through release of d-limonene. In the case where herbivory by larvae of the weevil *D. abbreviatus* was coincident with stimulation of the salicylic acid pathway, entomopathogenic nematode response was attenuated in this investigation. This interaction suggests a possible case of crosstalk between plant defense pathways. Insect herbivory has been shown in many instances to stimulate the jasmonic acid pathway [2, 14]. The jasmonic acid pathway, when stimulated, can antagonistically interact with the salicylic acid pathway, in some cases shutting down plant defense response [14].

While the jasmonic acid pathway is traditionally associated with plant responses to herbivory, stimulation of the salicylic acid pathway is often associated with defense against biotrophic pathogens [14]. In this case, its role in recruiting natural enemies may seem counter intuitive. Indeed the evolution and advantages of such attraction remain to be explored. One possible explanation is that the citrus-*D. abbreviatus*-entomopathogenic nematode interaction is not a simple closed system. There is a fourth, and prominent, player. The oomycete *Phytophthora* is frequently found in association with *D. abbreviatus* herbivory. Wounding of plant roots by *D. abbreviatus* opens a passage for infection by *Phytophthora* causing much greater damage to citrus trees and other plants than weevil herbivory alone [40]. The *Phytophthora*-*Diaprepes* weevil system is a complex that must be considered when developing management strategies for commercial citrus and plant production [41]. Because *Phytophthora* infections frequently accompany belowground weevil herbivory, recruitment of entomopathogenic nematodes by stimulation of the salicylic acid pathway may be an effective response for defense against attack by both an insect herbivore and a phytopathogen. We are currently exploring this hypothesis.
